# The Importance of Culture Change Associated With Novel Surgical Approaches and Innovation: Does Perioperative Care Transcend Technical Considerations for Pulmonary Lobectomy?

**DOI:** 10.3389/fsurg.2021.597410

**Published:** 2021-05-04

**Authors:** Kathrin Freystaetter, Benjamin R. Waterhouse, Nicholas Chilvers, Jason Trevis, Jonathan Ferguson, Ian Paul, Joel Dunning

**Affiliations:** Department of Cardiothoracic Surgery, The James Cook University Hospital, Middlesbrough, United Kingdom

**Keywords:** minimally invasive surgery, robotic surgery, innovation, lobectomy, thoracic surgery

## Abstract

Robotic thoracic surgery for pulmonary lobectomy was introduced at our unit in 2015, along with enhanced perioperative patient care pathways. We evaluated the effect of this practice change on short-term outcomes. Data on all adult patients who underwent a lobectomy in our unit between 2015 and 2019 were obtained retrospectively from our surgical database. Patients fell into three groups: conventional open surgery via thoracotomy, video-assisted thoracoscopic surgery (VATS), and robot-assisted thoracoscopic surgery (RATS). Survival was defined as survival to discharge. Our cohort included 722 patients. Three hundred and ninety-two patients (54.3%) underwent an open operation, 259 patients (35.9%) underwent VATS surgery, and 71 patients (9.8%) underwent a robotic procedure. Comparing these surgical approaches, there was no statistically significant difference in the overall incidence of post-operative complications (*p* = 0.15) as well as the incidence of wound infections, arrhythmias, prolonged air leaks, respiratory failure, or ICU readmissions. Additionally, there was no statistically significant difference in survival to discharge (*p* = 0.66). However, patients who had a VATS procedure were less likely to develop a post-operative chest infection (*p* = 0.01). Evaluating our practice over time, we found a decrease in the overall incidence of post-operative complications (*p* = 0.01) with an improvement in survival to discharge (*p* = 0.02). In our experience, VATS lobectomy was associated with a lower incidence of post-operative chest infections. However, the limitations of our study must be considered; factors such as patient selection that may have had a substantial impact. The culture change associated with adoption of a VATS and robotic surgical programme appears to have corresponded with an improved survival to discharge for all lobectomy patients, irrespective of surgical approach. Perioperative care may therefore have a more significant impact on outcomes than technical considerations.

## Introduction

The application of robotic surgical systems for lobectomies was first described in 2002 (1). Since then, robotic lobectomies have become increasingly utilized for the treatment of lung cancer. Particularly the rapid evolution of technology and enhanced user-friendliness have contributed to many centers initiating robotic surgical programmes (2). Outcomes are promising, with robotic thoracic surgical procedures being associated with improved survival, reduced length of stay in hospital, and fewer overall complications than conventional open lung resections (3). There are, however, significant barriers to robotic-assisted thoracic surgery, including an extensive learning curve, requirement for vast financial investment, as well as potential safety concerns as the unscrubbed operating surgeon is controlling the robotic system at the console away from the patient's bedside (4).

Robotic lobectomies were introduced in our unit in 2015 to complement our already well-established thoracic surgical practice consisting of routine video-assisted thoracoscopic surgery (VATS) and conventional open lobectomies. We viewed the implementation of this novel surgical technique as an impetus to update and supplement existing patient care pathways with additional perioperative measures such as proactive pain management, intensive physiotherapy, and an enhanced recovery after surgery (ERAS) protocol established in accordance with European Society of Thoracic Surgeons (ESTS) guidelines (5). These modernized patient care pathways were applied to all our thoracic surgical patients from 2015 onwards, irrespective of surgical approach.

We believe that such culture change on the back of state-of-the-art surgical procedures can improve overall patient care and therefore our aim was to assess whether all lobectomy patients benefit from such innovation. Consequently, we set out to evaluate the impact of surgical approaches on pulmonary lobectomy outcomes, taking perioperative care into account.

## Patients and Methods

This study was a retrospective, single center analysis including all adult patients undergoing lobectomy at our institution between 2015 and 2019. Patient data was collected from our prospectively-populated electronic surgical database. Our institution does not require Institutional Review Board or Ethics Committee approval for studies derived from anonymized institutional database data.

Survival was defined as survival to discharge following the primary operation. Patients were categorized by surgical access: conventional open surgery, VATS surgery, or robotic surgery. Additionally, patients were grouped by year of surgery to evaluate our practice over time.

We assessed patient characteristics, procedural data, post-operative complications, length of hospital stay, and survival to discharge. Continuous data is presented as mean and standard deviation or median and interquartile range (IQR) for normal and non-normal distributions, respectively. Categorical data is presented as the number and percentage. The Chi-Square Test was used for the analysis of categorical variables; Students' *t*-test and ANOVA were used for the analysis of continuous variables. SPSS statistical software version 23 (SPSS Inc., IBM, Chicago, Illinois, USA) was used for all statistical analyses, and *p* < 0.05 was deemed as statistically significant.

## Results

Seven hundred and twenty-two adult patients who underwent lobectomy were included in our analysis. Overall, the majority of patients had open surgery (*n* = 392, 54.3%), followed by VATS surgery (*n* = 259, 35.9%) and robotic surgery (*n* = 71, 9.8%). Comparing baseline characteristics for these groups (Table 1), patients who had open surgery were less likely to be diabetic and had a better performance status than those who had VATS or robot-assisted surgery. Patients who had robotic surgery were more likely to have ischemic heart disease and were more likely to be on long-term anticoagulant medication than those having open or VATS surgery. Over the 5-year time period studied (Table 2), we found a statistically significant variability in age (median age 67.5 in 2019 compared to 69.0 in 2015, *p* = 0.01) and gender (56.1% female in 2019 vs. 45.0% female in 2015, *p* = 0.01).

**Table 1:** Baseline patient characteristics for alternative operative approaches.

**Preoperative characteristics**	**Open approach**	**VATS approach**	**Robotic approach**	***P*-value**
Age (years)	71.0 (±11.0)	69.0 (±11.0)	70.0 (±10.3)	0.81
Female gender	203 (51.8%)	150 (57.9%)	42 (59.2%)	0.22
Height (cm)	165.2 (±21.0)	165.7 (±9.5)	164.8 (±10.8)	0.90
Weight (kg)	74.0 (±18.6)	76.0 (±24.5)	68.5 (±30.5)	0.67
Hypertension	191 (48.7%)	117 (45.2%)	39 (54.9%)	0.32
Insulin-dependent diabetes mellitus	4 (1.0%)	13 (5.0%)	3 (4.2%)	**0.01**
Ischaemic heart disease	54 (13.8%)	43 (16.6%)	21 (29.6%)	**<0.01**
Peripheral vascular disease	31 (7.9%)	14 (5.4%)	7 (9.9%)	0.32
Preoperative anticoagulation	16 (4.1%)	14 (5.4%)	8 (11.3%)	**0.04**
Previous stroke	29 (7.4%)	15 (5.8%)	5 (7.0%)	0.73
Preoperative creatinine (μmol/L)	69.0 (±21.5)	68.0 (±16.0)	75.5 (±25.3)	0.10
Preoperative hemoglobin (g/L)	134.0 (±16.0)	135 (±19.5)	138.0 (±17.0)	0.26
Previous history of cancer	90 (23.0%)	65 (25.1%)	26 (36.6%)	0.05
Smoking history				
Never smoker	54 (13.8%)	35 (13.5%)	14 (20.3%)	
Ex-smoker	245 (62.8%)	160 (61.8%)	43 (62.3%)	
Current smoker	91 (23.3%)	64 (24.7%)	12 (17.4%)	0.53
Chronic pulmonary disease	164 (41.8%)	118 (45.6%)	28 (39.4%)	0.53
Steroid therapy	8 (2.0%)	2 (0.8%)	2 (2.8%)	0.31
Preoperative lung function				
Measured FEV1 (L)	1.9 (±1.2)	2.0 (±0.9)	1.6 (±0.70)	0.19
Predicted FEV1 (%)	84.1 (±21.5)	85.4 (±21.6)	83.3 (±22.1)	0.66
Measured FVC (L)	3.0 (±1.5)	3.1 (±1.4)	2.7 (±1.4)	0.19
Predicted FVC (%)	101.0 (±26.0)	101.0 (±22.0)	97.5 (±40.0)	0.93
TLCO predicted (%)	69.9 (±20.0)	68.7 (±21.5)	66.2 (±21.9)	0.65
KCO predicted (%)	77.0 (±30.0)	73.0 (±31.0)	66.0 (±34.0)	0.17
Performance status				
0	176 (44.9%)	82 (31.7%)	21 (29.9%)	
1	202 (51.5%)	161 (62.2%)	43 (60.6%)	
2	11 (2.8%)	16 (6.2%)	6 (8.5%)	
3	1 (0.3%)	0 (0.0%)	1 (1.4%)	
4	2 (0.5%)	0 (0.0%)	0 (0.0%)	**<0.01**
ASA grade				
1	7 (1.8%)	4 (1.5%)	4 (5.6%)	
2	145 (37.0%)	93 (35.9%)	20 (28.2%)	
3	233 (59.4%)	154 (59.5%)	46 (64.8%)	
4	7 (1.8%)	8 (3.1%)	1 (1.4%)	0.81

*FEV1, Forced expiratory volume in 1 s; FVC, forced vital capacity; TLCO, transfer factor for carbon monoxide; KCO, carbon monoxide transfer coefficient; ASA, American Society of Anesthesiologists Classification. The bold values are deemed statistically significant (i.e., p < 0.05), these were written in bold to highlight statistically significant findings for the reader's ease*.

**Table 2:** Baseline patient characteristics over time.

**Preoperative characteristics**	**2015**	**2016**	**2017**	**2018**	**2019**	***P*-value**
Age (years)	69.0 (±8.0)	70.0 (±8.0)	70.0 (±9.0)	71.5 (±13.0)	67.5 (±13.8)	**0.01**
Female gender	59 (45.0%)	78 (51.7%)	98 (65.3%)	72 (54.1%)	88 (56.1%)	**0.01**
Height (cm)	166.0 (±9.0)	170.5 (±18.5)	163.0 (±13.5)	165.0 (±15.0)	166.0 (±13.5)	0.13
Weight (kg)	77.9 (±18.2)	74.0 (±15.7)	75.2 (±16.7)	72.7 (±15.4)	71.8 (±25.8)	0.07
Hypertension	60 (45.8%)	63 (41.7%)	63 (42.0%)	84 (63.2%)	77 (49.0%)	**<0.01**
Insulin-dependent diabetes mellitus	4 (3.1%)	5 (3.3%)	3 (2.0%)	4 (3.0%)	4 (2.5%)	0.96
Ischaemic heart disease	17 (13.0%)	33 (21.9%)	27 (18.0%)	20 (15.0%)	21 (13.4%)	0.21
Peripheral vascular disease	6 (4.6%)	10 (6.6%)	7 (4.7%)	12 (9.0%)	17 (10.8%)	0.16
Preoperative anticoagulation	4 (3.1%)	5 (3.3%)	10 (6.7%)	13 (9.8%)	6 (3.08%)	0.06
Previous stroke	6 (4.6%)	10 (6.6%)	11 (7.3%)	15 (11.3%)	7 (4.5%)	0.15
Preoperative creatinine (μmol/L)	72.0 (±27.0)	74.5 (±26.5)	65.0 (±18.5)	68.0 (±17.3)	70.5 (±22.0)	**0.01**
Preoperative hemoglobin (g/L)	136.9 (±15.1)	133.2 (±16.4)	134.0 (±13.2)	132.8 (±14.3)	134.2 (±15.5)	0.19
Previous history of cancer	36 (27.5%)	32 (21.2%)	41 (27.3%)	31 (23.3%)	41 (26.1%)	0.68
Smoking history						
Never smoker	12 (9.2%)	17 (11.4%)	34 (22.7%)	12 (9.0%)	28 (18.1%)	
Ex-smoker	88 (67.2%)	94 (63.1%)	78 (52.0%)	91 (68.4%)	97 (62.6%)	
current smoker	31 (23.7%)	38 (25.5%)	38 (25.3%)	30 (22.6%)	30 (19.4%)	**0.01**
Chronic pulmonary disease	51 (38.9%)	74 (49.0%)	59 (39.3%)	70 (52.6%)	56 (35.7%)	**0.02**
Steroid therapy	0 (0.0%)	5 (3.3%)	2 (1.3%)	3 (2.3%)	2 (1.3%)	0.14
Preoperative lung function						
Measured FEV1 (L)	2.0 (±1.1)	1.8 (±0.9)	1.9 (±1.0)	1.7 (±0.7)	2.1 (±1.2)	0.31
Predicted FEV1 (%)	76.0 (±36.0)	74.5 (±27.5)	88.0 (±33.0)	83.0 (±27.3)	86.5 (±27.3)	0.15
Measured FVC (L)	3.1 (±0.8)	3.0 (±0.8)	3.0 (±0.9)	2.9 (±0.8)	3.1 (±0.9)	0.63
Predicted FVC (%)	97.0 (±18.0)	95.0 (±28.0)	108.0 (±28.0)	100.5 (±29.0)	104.5 (±24.0)	0.12
TLCO predicted (%)	73.1 (±21.8)	64.1 (±19.0)	69.7 (±20.9)	68.8 (±20.9)	68.4 (±20.7)	0.52
KCO predicted (%)	78.0 (±29.0)	68.5 (±26.0)	74.0 (±34.0)	72.5 (±25.0)	77.0 (±29.0)	0.80
Performance status						
0	50 (38.2%)	56 (37.1%)	64 (42.7%)	45 (33.8%)	64 (40.8%)	
1	77 (58.8%)	85 (56.3%)	77 (51.3%)	83 (62.4%)	84 (53.5%)	
2	4 (3.1%)	9 (6.0%)	8 (5.3%)	5 (3.8%)	7 (4.5%)	
3	0 (0.0%)	1 (0.1%)	1 (0.7%)	0 (0.0%)	0 (0.0%)	
4	0 (0.0%)	0 (0.0%)	0 (0.0%)	0 (0.0%)	2 (1.3%)	0.76
ASA grade						
1	4 (3.1%)	4 (2.6%)	0 (0.0%)	0 (0.0%)	7 (4.5%)	
2	35 (26.7%)	51 (33.8%)	71 (47.3%)	46 (34.6%)	55 (35.0%)	
3	87 (66.4%)	91 (60.3%)	79 (52.7%)	86 (64.7%)	90 (57.3%)	
4	5 (3.8%)	5 (3.3%)	0 (0.0%)	1 (0.8%)	5 (3.2%)	**0.04**

*FEV1, Forced expiratory volume in 1 s; FVC, forced vital capacity; TLCO, transfer factor for carbon monoxide; KCO, carbon monoxide transfer coefficient; ASA, American Society of Anesthesiologists Classification. The bold values are deemed statistically significant (i.e., p < 0.05), these were written in bold to highlight statistically significant findings for the reader's ease*.

Our unit's procedural data are shown in Tables 3, 4. We found that the percentage of open procedures remained fairly constant while the percentage of robotic procedures per year increased at the cost of VATS procedures (*p* < 0.01). The majority of lobectomies were performed for malignancy. Robot-assisted lobectomies were more likely to be left lower or right lower lobectomies, while the distribution was similar for VATS and open cases. Most procedures were uneventful and the majority of patients were transferred to the high dependency unit (HDU) for initial post-operative care. Patients that required admission to the intensive care unit were more likely to have undergone an open procedure (*p* = 0.01).

**Table 3:** Procedural data per surgical approach.

**Procedural data**	**Open approach**	**VATS approach**	**Robotic approach**	***P*-value**
Total procedures				
2015	55 (42.0%)	68 (51.9%)	8 (6.1%)	
2016	81 (53.6%)	57 (37.7%)	13 (8.6%)	
2017	91 (60.7%)	49 (32.7%)	10 (6.7%)	
2018	77 (57.9%)	42 (31.6%)	14 (10.5%)	
2019	88 (56.1%)	43 (27.4%)	26 (16.6%)	**<0.01**
Indication				
Malignancy	368 (55.8%)	229 (34.7%)	62 (9.4%)	
Benign disease	24 (38.1%)	30 (47.6%)	9 (14.3%)	**0.03**
Type of procedure				
Left upper lobectomy	93 (57.4%)	63 (38.9%)	6 (3.7%)	**0.01**
Left lower lobectomy	55 (48.7%)	36 (31.9%)	22 (19.5%)	**<0.01**
Right upper lobectomy	126 (57.5%)	80 (36.5%)	13 (6.0%)	0.06
Right middle lobectomy	43 (53.8%)	30 (37.5%)	7 (8.8%)	0.91
Right lower lobectomy	91 (57.6%)	43 (27.2%)	24 (15.2%)	**0.01**
Conversion to thoracotomy				
No	N/A	214 (77.0%)	64 (23.0%)	
Planned conversion	N/A	24 (100.0%)	0 (0.0%)	
Technical difficulty	N/A	15 (88.2%)	2 (11.8%)	
Controlled bleeding	N/A	5 (62.5%)	3 (37.5%)	
Uncontrolled bleeding	N/A	1 (33.3%)	2 (66.7%)	**<0.01**
Post-operative destination				
Ward	3 (100.0%)	0 (0.0%)	0 (0.0%)	
HDU	380 (53.6%)	259 (36.5%)	70 (9.9%)	
CICU	9 (90.0%)	0 (0.0%)	1 (10.0%)	**0.01**

*HDU, high dependency unit; CICU, cardiothoracic intensive care unit. The bold values are deemed statistically significant (i.e., p < 0.05), these were written in bold to highlight statistically significant findings for the reader's ease*.

**Table 4:** Procedural data over time.

**Procedural data**	**2015**	**2016**	**2017**	**2018**	**2019**	***P*-value**
Access						
Open approach	55 (42.0%)	81 (53.6%)	91 (60.7%)	77 (57.9%)	88 (56.1%)	
VATS approach	68 (51.9%)	57 (37.7%)	49 (32.7%)	42 (31.6%)	43 (27.4%)	
Robotic approach	8 (6.1%)	13 (8.6%)	10 (6.7%)	14 (10.5%)	26 (16.6%)	**<0.01**
Indication						
Malignancy	113 (86.3%)	140 (92.7%)	142 (94.7%)	122 (91.7%)	142 (90.4%)	
Benign disease	18 (13.7%)	11 (7.3%)	8 (5.3%)	11 (8.3%)	15 (9.6%)	0.14
Type of procedure						
Left upper lobectomy	27 (20.6%)	39 (25.8%)	39 (26.0%)	26 (19.5%)	31 (19.1%)	0.46
Left lower lobectomy	22 (16.8%)	19 (12.6%)	23 (15.3%)	22 (16.5%)	27 (17.2%)	0.81
Right upper lobectomy	39 (29.8%)	47 (31.1%)	43 (28.7%)	43 (32.3%)	47 (29.9%)	0.97
Right middle lobectomy	17 (13.0%)	17 (11.3%)	13 (8.7%)	18 (13.5%)	15 (9.6%)	0.64
Right lower lobectomy	26 (19.8%)	32 (21.2%)	35 (23.3%)	29 (21.8%)	36 (22.9%)	0.96
Conversion to thoracotomy						
No	66 (86.8%)	56 (80.0%)	50 (84.7%)	49 (87.5%)	57 (82.6%)	
Planned conversion	7 (9.2%)	7 (10.0%)	6 (10.2%)	2 (3.6%)	2 (2.9%)	
Technical difficulty	2 (2.6%)	4 (5.7%)	1 (1.7%)	4 (7.1%)	6 (8.7%)	
Controlled bleeding	1 (1.3%)	1 (1.4%)	2 (3.4%)	0 (0.0%)	4 (5.8%)	
Uncontrolled bleeding	0 (0.0%)	2 (2.9%)	0 (0.0%)	1 (1.8%)	0 (0.0%)	0.13
Post-operative destination						
Ward	1 (0.8%)	1 (0.7%)	0 (0.0%)	0 (0.0%)	1 (0.6%)	
HDU	128 (97.7%)	149 (98.7%)	150 (100.0%)	130 (97.7%)	152 (96.8%)	
CICU	2 (1.5%)	1 (0.7%)	0 (0.0%)	3 (2.3%)	4 (2.5%)	0.54

*HDU, high dependency unit; CICU, cardiothoracic intensive care unit. The bold values are deemed statistically significant (i.e., p < 0.05), these were written in bold to highlight statistically significant findings for the reader's ease*.

The incidence of in-hospital post-operative complications is shown in Tables 5, 6. Patients in the VATS group were less likely to develop a chest infection compared to the open or robotic group (*p* = 0.01). We could not identify a statistically significant difference in the incidence of any other recorded post-operative complications comparing the different surgical approaches, including survival to discharge. Comparing in-hospital outcomes over time, we found a decrease in the incidence of overall post-operative complications (*p* = 0.01), while there was an increase in post-operative urinary retention (*p* = 0.01). Survival to discharge improved with increasing experience (*p* = 0.02) while there was no statistically significant difference in post-operative length of hospital stay (*p* = 0.40).

**Table 5:** Post-operative complications per surgical approach.

**Outcomes**	**Open approach**	**VATS approach**	**Robotic approach**	***P*-value**
Any complication	144 (36.7%)	77 (29.7%)	27 (38.0%)	0.15
Chest infection	87 (22.2%)	33 (12.7%)	14 (19.7%)	**0.01**
Wound infection	2 (0.5%)	0 (0.0%)	0 (0.0%)	0.29
Urinary tract infection	6 (1.5%)	1 (0.4%)	2 (2.8%)	0.18
*C. difficile* diarrhea	4 (1.0%)	0 (0.0%)	0 (0.0%)	0.09
Post-operative atrial fibrillation	29 (7.4%)	14 (5.4%)	4 (5.6%)	0.57
Respiratory failure	7 (1.8%)	7 (2.7%)	2 (2.8%)	0.69
Acute kidney injury	4 (1.0%)	6 (2.3%)	1 (1.4%)	0.43
Urinary retention	12 (3.1%)	4 (1.5%)	2 (2.8%)	0.47
Gastrointestinal complications	5 (1.3%)	3 (1.2%)	0 (0.0%)	0.43
Cerebrovascular accident	1 (0.3%)	0 (0.0%)	0 (0.0%)	0.54
Surgical emphysema	4 (1.0%)	7 (2.7%)	0 (0.0%)	0.09
Chylothorax	2 (0.5%)	0 (0.0%)	1 (1.4%)	0.20
Peripheral limb ischaemia	1 (0.3%)	0 (0.0%)	0 (0.0%)	0.54
Vocal cord palsy	1 (0.3%)	0 (0.0%)	0 (0.0%)	0.54
CICU admission	12 (3.1%)	11 (4.2%)	2 (2.8%)	0.69
Persistent air leak >7 days	31 (7.9%)	29 (11.2%)	4 (5.6%)	0.21
IPPV	1 (0.3%)	5 (1.9%)	1 (1.4%)	0.08
Return to theater	9 (2.3%)	4 (1.5%)	1 (1.4%)	0.75
Median length of hospital stay	4.0 (±6.0)	4.0 (±7.0)	5.5 (±14.25)	0.12
Survival to discharge	381 (97.2%)	254 (98.1%)	70 (98.6%)	0.66

*CICU, cardiothoracic intensive care unit; IPPV, intermittent positive pressure ventilation. The bold values are deemed statistically significant (i.e., p < 0.05), these were written in bold to highlight statistically significant findings for the reader's ease*.

**Table 6:** Post-operative complications over time.

**Outcomes**	**2015**	**2016**	**2017**	**2018**	**2019**	***P*-value**
Any complication	34 (26.0%)	58 (38.4%)	57 (38.0%)	56 (42.1%)	43 (27.4%)	**0.01**
Chest infection	21 (16.0%)	38 (25.2%)	24 (16.0%)	29 (21.8%)	22 (14.0%)	0.07
Wound infection	0 (0.0%)	0 (0.0%)	1 (0.7%)	1 (0.8%)	0 (0.0%)	0.44
Urinary tract infection	0 (0.0%)	2 (1.3%)	2 (1.3%)	3 (2.3%)	2 (1.3%)	0.38
*C. difficile* diarrhea	2 (1.5%)	0 (0.0%)	1 (0.7%)	1 (0.8%)	0 (0.0%)	0.28
Post-operative atrial fibrillation	7 (5.3%)	7 (4.6%)	14 (9.3%)	11 (1.5%)	8 (5.1%)	0.36
Respiratory failure	1 (0.8%)	7 (4.6%)	1 (0.7%)	4 (3.0%)	3 (1.9%)	0.11
Acute kidney injury	0 (0.0%)	2 (1.3%)	4 (2.7%)	2 (1.5%)	3 (1.9%)	0.26
Urinary retention	0 (0.0%)	1 (0.7%)	5 (3.3%)	6 (4.5%)	6 (3.8%)	**0.01**
Gastrointestinal complications	3 (2.3%)	1 (0.7%)	1 (0.7%)	3 (2.3%)	0 (0.0%)	0.16
Cerebrovascular accident	0 (0.0%)	0 (0.0%)	0 (0.0%)	1 (0.8%)	0 (0.0%)	0.50
Surgical emphysema	2 (1.5%)	1 (0.7%)	4 (2.7%)	3 (2.3%)	1 (0.6%)	0.48
Chylothorax	0 (0.0%)	1 (0.7%)	0 (0.0%)	0 (0.0%)	2 (1.3%)	0.25
Peripheral limb ischaemia	0 (0.0%)	1 (0.7%)	0 (0.0%)	0 (0.0%)	0 (0.0%)	0.54
Vocal cord palsy	0 (0.0%)	0 (0.0%)	0 (0.0%)	1 (0.8%)	0 (0.0%)	0.50
CICU readmission	4 (3.1%)	8 (5.3%)	3 (2.0%)	7 (5.3%)	3 (1.9%)	0.29
Persistent air leak >7 days	12 (9.2%)	14 (9.3%)	15 (10.0%)	16 (12.0%)	7 (4.5%)	0.22
IPPV	2 (1.5%)	4 (2.6%)	0 (0.0%)	1 (0.8%)	0 (0.0%)	0.05
Return to theater	1 (0.8%)	5 (3.2%)	2 (1.3%)	3 (2.3%)	3 (1.9%)	0.58
Median length of hospital stay	5 (±7.0)	8.5 (±13.25)	4.0 (±5.0)	5.0 (±7.75)	3.5 (±5.0)	0.40
Survival to discharge	129 (98.5%)	146 (96.7%)	147 (98.0%)	126 (94.7%)	157 (100.0%)	**0.02**

*CICU, cardiothoracic intensive care unit; IPPV, intermittent positive pressure ventilation. The bold values are deemed statistically significant (i.e., p < 0.05), these were written in bold to highlight statistically significant findings for the reader's ease*.

## Discussion

Numerous studies have compared post-operative outcomes of patients undergoing pulmonary lobectomy via open or minimally invasive surgical procedures including VATS, or robot-assisted surgery.

Early reports have established the advantages of VATS approaches, demonstrating improved pain control and reduced length of stay compared to open surgery, while also maintaining similar survival rates (6). However, not all studies have confirmed these initial results. Gopaldas et al. analyzed a cohort of more than 13,000 patients and found that patients in the VATS group were more likely to experience an intraoperative complication compared to those patients that had a thoracotomy for their resection, which the authors link to the learning curve of novel approaches (7), which must be taken into account when interpreting unit data such as this.

Robotic surgical programmes have become more commonplace due to the recognized safety and feasibility of these procedures. Analysis of early experiences performing robotic lobectomies demonstrated similar rates of post-operative complications with a shorter hospital stay in patients who had a robotic procedure compared to thoracotomy (8). Cerfolio et al. reported adequate lymph node resection and observed benefits of robot-assisted surgery including reduced length of stay, morbidity, and mortality (9). Furthermore, multi-center reviews have suggested that the robotic approach provides similar long-term survival of up to 5 years compared to VATS and open surgery (10).

Comparing the robotic approach to VATS procedures, similar results are reported for intraoperative and short-term post-operative outcomes including blood loss and length of hospital stay, although patients in the robotic group reported a faster return to usual activities and stopped narcotics earlier than those in the VATS group (11). A significantly shorter hospital stay following robotic lobectomy was also reported by Jang et al. (12). However, Augustin et al. reported significant advantages of the VATS approach compared to robotic surgery, including reduced blood loss as well as a financial benefit in this cohort (13).

These somewhat conflicting results prompted us to evaluate our own practice. We observed a lower incidence of post-operative chest infections in VATS lobectomy patients compared to open and robotic procedures, which one might postulate related to improved mobility in the early post-operative phase, but we do not have specific data to support this. However, the incidence of other post-operative complications as well as length of hospital stay and survival to discharge was similar across all three groups. Analyzing our unit's experience over time following the introduction of robot-assisted thoracic surgery in 2015, we have been able to significantly reduce the overall incidence of post-operative complications and increase short-term survival for all lobectomy patients, irrespective of surgical approach.

We believe that this substantial improvement over time is not only linked to the operating surgeon's learning curve and development of technical skills, but also to the optimization of post-operative care by the multi-disciplinary team on the ward. The introduction of the robotic programme came hand-in-hand with the initiation of the ERAS protocol, including proactive pain management with alternative treatment measures such as acupuncture, early mobilization, intensive physiotherapy, and expedited chest drain removal. Although linked to the expansion of our minimally-invasive surgical programme, this protocol was standardized and applied to all thoracic surgical patients including conventional approaches.

In our study we found a decrease in the number of VATS lobectomies performed per year since introduction of the robotic programme. Our unit employs three thoracic surgeons, two of which perform minimally-invasive lobectomies (i.e., VATS and robot-assisted surgery), while the third performs only open lobectomies, which is likely to account for this finding. We expect the number of VATS lobectomies to continue decreasing in the future as we increase access to the robotic theater and thus robot-assisted thoracoscopic surgery (RATS) potentially becoming the new standard surgical approach in our unit, with open lobectomies reserved for technically challenging cases.

In our unit, initial post-operative care is usually done on our cardiothoracic HDU. Planned post-operative admission to our cardiothoracic intensive care unit (CICU) is reserved for patients with significant comorbidities such as heart failure, or for technically challenging procedures such as large or proximal resections or sleeve resections. In these cases, the operating surgeon may elect to perform the lobectomy via an open approach to prevent a prolonged operative time or intraoperative complications, which may account for the observed increased incidence of thoracotomy patients admitted to CICU.

The majority of conversions from minimally-invasive approaches to thoracotomy occurred in the context of technically challenging VATS cases, most conversions being anticipated and planned. In two instances, technical difficulties led to conversion from RATS to open lobectomy. In the overall time period assessed, only six VATS (2.3%) and five RATS (7.0%) cases were converted to open due to intraoperative bleeding complications. The observed VATS to open conversion rate is in line with that reported by other units; the RATS to open conversion rate may have been slightly increased due to the initial learning curve of the surgeons. Analysis of the conversion rate over time demonstrates a reduction in planned conversions, suggesting that cases that were anticipated to be challenging, were adequately planned to receive a thoracotomy approach. The rate of conversion due to intraoperative technical difficulties decreased initially and then increased, which may be linked to surgeons' confidence levels over time and attempting more challenging cases with minimally invasive approaches. However, the conversion rate due to bleeding remains low in our unit and represents a recognized complication of minimally invasive surgery, which is not totally eliminable.

The major limitation of our study is its retrospective, single-center design. Furthermore, an inadvertent patient selection bias will be present, particularly during the early days of the robotic surgical programme where choosing favorable patients for the initial steep phase of the learning curve is to be expected.

## Conclusion

The culture change associated with the expansion of our VATS, as well as institution of a robotic surgical programme, was associated with reduced overall post-operative complications, reduced chest infections, and improved survival to discharge from hospital. Introducing innovative practice therefore has significant potential in improving outcomes for all thoracic surgical patients and highlights the importance of a well-functioning multidisciplinary team. Perioperative patient care may thus be more significant in determining short-term outcomes than technical considerations, with further studies required to define causality.

## Data Availability Statement

The raw data supporting the conclusions of this article will be made available by the authors, without undue reservation.

## Ethics Statement

Ethical review and approval was not required for the study on human participants in accordance with the local legislation and institutional requirements. Written informed consent for participation was not required for this study in accordance with the national legislation and the institutional requirements.

## Author Contributions

KF: investigation, methodology, formal analysis, and writing—original draft. NC, BW, and JT: investigation, writing—review, and editing. IP, JF, and JD: conceptualization, supervision, writing—review, and editing. All authors contributed to the article and approved the submitted version.

## Conflict of Interest

The authors declare that the research was conducted in the absence of any commercial or financial relationships that could be construed as a potential conflict of interest.
